# The blood oxygen level dependent (BOLD) effect of in-vitro myoglobin and hemoglobin

**DOI:** 10.1038/s41598-021-90908-x

**Published:** 2021-06-01

**Authors:** Dominik P. Guensch, Matthias C. Michel, Stefan P. Huettenmoser, Bernd Jung, Patrik Gulac, Adrian Segiser, Sarah L. Longnus, Kady Fischer

**Affiliations:** 1 Department of Anaesthesiology and Pain Medicine, Inselspital, Bern University Hospital, University of Bern, Bern, Switzerland; 2 Department of Diagnostic, Interventional and Paediatric Radiology, Inselspital, Bern University Hospital, University of Bern, Bern, Switzerland; 3 Department of Cardiovascular Surgery, Inselspital, Bern University Hospital, University of Bern , Bern, Switzerland; 4 Department for BioMedical Research, University of Bern, Bern, Switzerland; 5 Department of Pharmacology and Toxicology, Faculty of Pharmacy, Comenius University, Bratislava, Slovakia

**Keywords:** Preclinical research, Cardiology, Magnetic resonance imaging

## Abstract

The presence of deoxygenated hemoglobin (Hb) results in a drop in T2 and T2* in magnetic resonance imaging (MRI), known as the blood oxygenation level-dependent (BOLD-)effect. The purpose of this study was to investigate if deoxygenated myoglobin (Mb) exerts a BOLD-like effect. Equine Met-Mb powder was dissolved and converted to oxygenated Mb. T1, T2, T2*-maps and BOLD-bSSFP images at 3Tesla were used to scan 22 Mb samples and 12 Hb samples at room air, deoxygenation, reoxygenation and after chemical reduction. In Mb, T2 and T2* mapping showed a significant decrease after deoxygenation (− 25% and − 12%, *p* < 0.01), increase after subsequent reoxygenation (+ 17% and 0% vs. room air, *p* < 0.01), and finally a decrease in T2 after chemical reduction (− 28%, *p* < 0.01). An opposite trend was observed with T1 for each stage, while chemical reduction reduced BOLD-bSSFP signal (− 3%, *p* < 0.01). Similar deflections were seen at oxygenation changes in Hb. The T1 changes suggests that the oxygen content has been changed in the specimen. The shortening of transverse relaxation times in T2 and T2*-mapping after deoxygenation in Mb specimens are highly indicative of a BOLD-like effect.

## Introduction

Ogawa et al. first described the utility of sequences exploiting the blood oxygen level-dependent (BOLD) effect in brain magnetic resonance imaging (MRI) scans for functional imaging^1^. Since then the technique has been used extensively for brain imaging^2^. In the last 20 years this technique has been adapted and extensively validated in cardiovascular magnetic resonance (CMR) studies, mainly to assess (inducible) ischemia^3^. However, only more recently has this paradigm been used in patient settings^3–7^. This so-called BOLD-effect uses deoxygenated hemoglobin (dHb) as endogenous contrast^2,3^. The presence of the paramagnetic dHb results in microscopic magnetic field inhomogeneities, which alters the bulk susceptibility of the solvent medium. This will lead to a decrease in T2 and moreover T2* relaxation times or a reduction in signal intensity (SI) in sequences sensitive to this BOLD effect. Oxygenated Hb (HbO_2_) in turn has weak effects on the local magnetic field and has a negligible effect on the relaxation times of the bulk water dissolving the molecules. The relative proportion of dHb on the total Hb content of the tissue determines T2, T2*, and SI, respectively. Because of its reliance on dHb, BOLD imaging is a reflection of the local oxygen supply and demand balance and provides a measure of vascular (dys-)function or ischaemia. In in-vivo, the signal attenuation in these images originates in the compartment of the post-capillary venules^8^. Mechanisms that increase dHb concentration, such as diminished perfusion and oxygen supply or increased oxygen extraction and workload attenuate local signal intensity (SI), whereas those reducing relative dHb concentration, such as increased blood flow beyond the oxygen demand (luxury-perfusion) will enhance SI and produce regional hyper-intensity^9^. In a healthy system, metabolites indicating increased workload and temporary desaturation will initiate a feedback mechanism to increase oxygen supply to maintain the local oxygen balance. However, in cases of vascular dysfunction, if an increase in workload and oxygen consumption cannot be matched by an increased blood supply, then local deoxygenation occurs consequently reducing BOLD based SI.

Myoglobin (Mb) is a monomeric molecule with a close homology to the alpha and beta sub-units of Hb and is ubiquitously found in varying concentrations in most striated muscle fibers and the myocardium^10^. Mb has a half-saturation pressure p_50_ of 2.8 mmHg^11^, while Hb’s p_50_ is approximately 25 mmHg. Thus, Mb has a greater affinity to O_2_ than Hb. Therefore, in the presence of normal blood oxygenation in the tissue, Mb is very unlikely to desaturate at resting conditions^10^ and it is thus more difficult to investigate in-vivo. However, in skeletal muscle Mb is known to desaturate to deoxygenated Mb (dMb) during increased muscle activity^12^ and restricted blood flow^13^. In the myocardium its importance is rather controversial^14–16^. Magnetic Resonance Spectroscopy (MRS) has been shown to be able to detect different concentrations of dMb in-vivo, however there are no studies reporting on the BOLD-effect of dMb. Due to the low p_50_ of Mb, studies assessing the changes in magnetic susceptibility of Mb can only be performed in absence of Hb for validation of this concept. This study aimed to assess if Mb has a BOLD-effect that can be detected by clinical cardiovascular magnetic resonance parametric mapping sequences.

## Results

### Presence and patency of myoglobin

**Figure 1:**
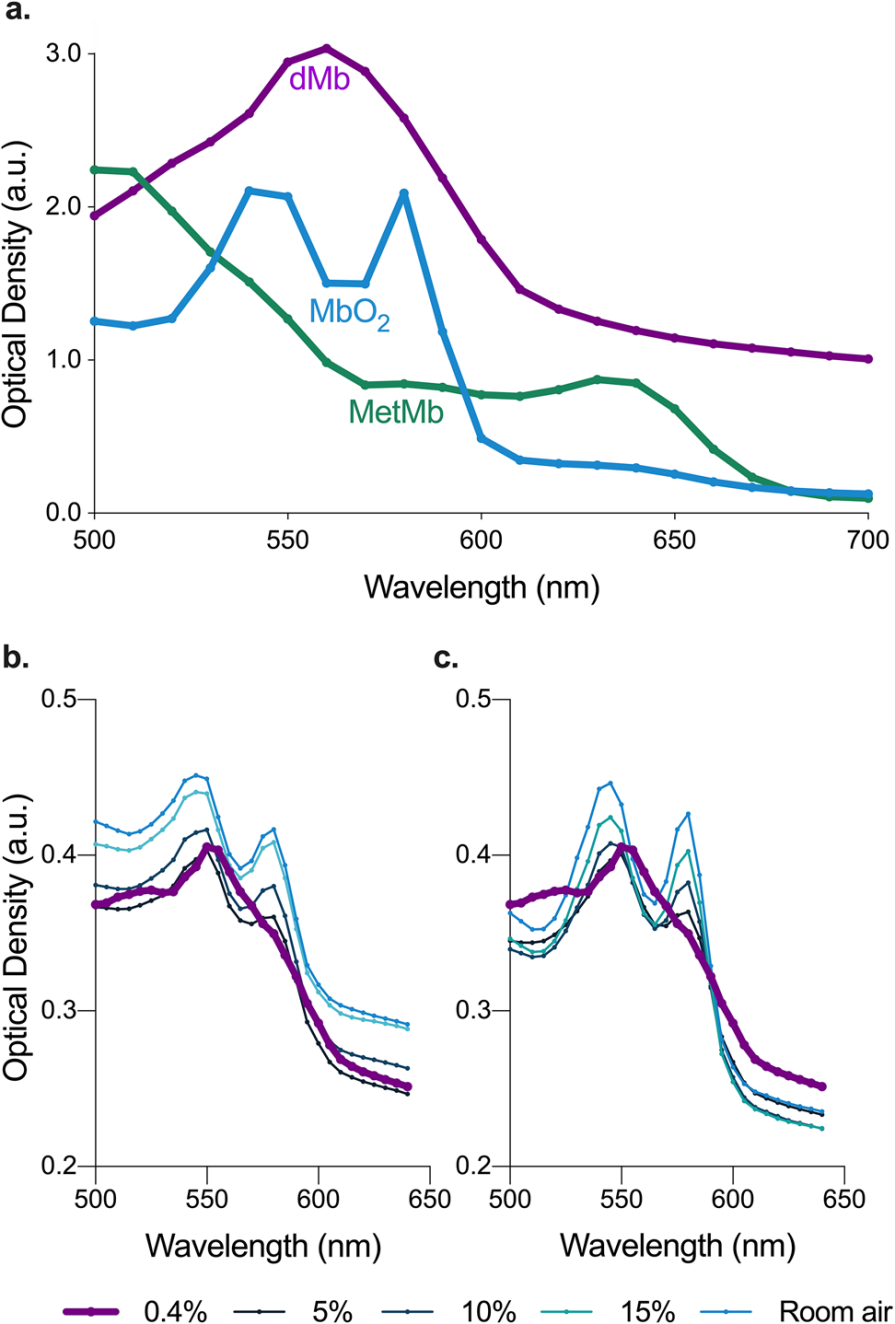
Light spectroscopy of myoglobin oxygenation: (**a**) Light spectroscopy verified the presence of oxygenated myoglobin (MbO_2_), by the double peak spectrum (blue) in comparison to deoxygenated myoglobin (dMb, purple) and the untreated stock-solution of equine methyl-Mb (metMb, green). (**b**) Light spectroscopy further showed the functional capacity of MbO_2_ to desaturate by reducing oxygen concentration from room air to 0.4%, and then its ability to re-oxygenate with the increase in oxygen content (**c**). Image prepared using Graphpad Prism v 8.0 (www.graphpad.com).

Equine metmyoglobin (metMb) powder was dissolved and was successfully converted to an oxygenated Mb (MbO_2_) solution through auto-oxygenation, shown by light-spectroscopy. Half the samples were measured with a standard protein concentration essay, yielding a concentration between 4.24 and 4.42 mg/ml after passing the desalting column. Figure 1a. shows the characteristic spectra of metMb (stock solution), dMb (after addition of sodium-dithionite) and MbO_2_ (after desalting the specimen from sodium-dithionite and subsequent auto-oxygenation) in our samples, which are in line with external published findings^11^. Figure 1b shows that both MbO_2_ peaks diminish with decreasing oxygen concentration in the plate reader. At 0.4% oxygen concentration the characteristic double-peak spectrum of MbO_2_ has vanished, and only the single peak spectrum remains, which is characteristic for dMb, demonstrating that no MbO_2_ remained in these specimens. Increasing ambient oxygen concentration resulted in a recovery of the MbO_2_ curve (Fig. 1c).

### Imaging results

**Figure 2:**
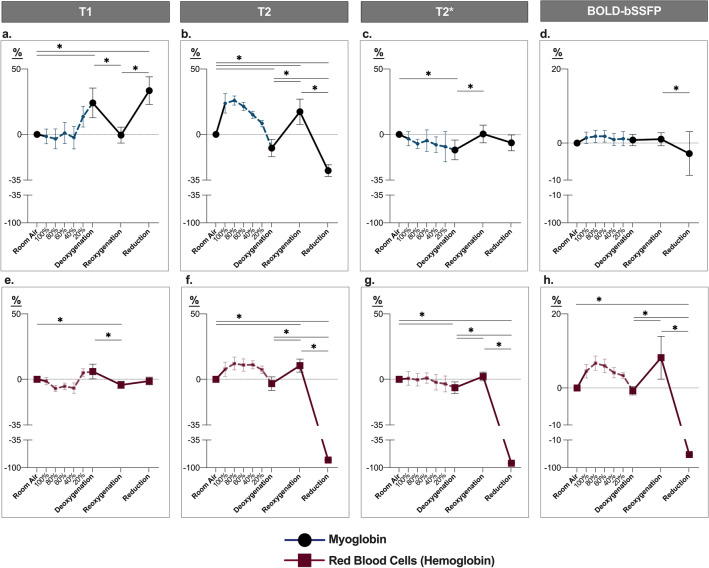
MRI detects the changing oxygenation of myoglobin and hemoglobin: Mean ± 95% confidence intervals of the percent-change from the room air level are shown for each oxygenation state when measured with a T1 map (**a**, **e**), a T2 map (**b**, **f**), a T2* map (**c**, **g**) and a blood oxygen level-dependent balanced steady-state free precession image (BOLD-bSSFP; **d**, **h**). The responses for isolated myoglobin are shown in the top row and red blood cells representing the response of hemoglobin in the bottom row. Dotted lines depict the stepwise de-oxygenation process from 100% O_2_/0% N_2_ to a mix of 0% O_2_/100% N_2_ in 20% increments. A break is present in the negative values of the y-axis to depict the significant reduction by chemical reduction in the same figure. Image prepared using Graphpad Prism v 8.0 (www.graphpad.com). *p < 0.05 represents a significant difference in signal between major oxygenation levels.

Twenty-two samples of Mb and twelve samples of Hb from packed red blood cells underwent MRI imaging. T1, T2, T2* mapping and BOLD-sensitive balanced steady-state free precession (bSSFP) cine images were acquired at room air, after deoxygenation by bubbling 100% nitrogen (N_2_), reoxygenation by bubbling 100% oxygen (O_2_) and after chemical reduction to dMb and dHb, respectively, using sodium dithionite. The relative relaxation time and signal intensity changes have been statistically assessed and are shown in Fig. 2. In twelve of these Mb and Hb samples, additional images were acquired at 20%, 40%, 60% and 80% oxygen concentration for visualization purposes. Due to poor image quality, 1% of the samples could not be analyzed for the T1 maps. along with 2% for both the T2 maps and BOLD-bSSFP cine. T2* maps had the poorest image quality with an 11% of the levels deemed to be unanalyzable due to artifact. Imaging measurements are shown as a response (%-change) in relaxation times and signal intensity from the first level acquired at room air (mean ± 95% confidence interval).

### T1 and T2 mapping

T1 Mapping revealed that deoxygenating the Mb samples (Fig. 2a) through N_2_-bubbling increased T1 relaxation time by 24 ± 11% (*p* < 0.01) over that of the auto-oxygenated state at room air. After O_2_-bubbling T1 relaxation time dropped down to that of baseline (− 0.6 ± 6%, *p* > 0.99 vs. baseline, *p* < 0.01 vs. deoxygenation). Similar to the deoxygenated Mb, the chemically reduced Mb solution also increased T1 above the room air and reoxygenated states (+ 34 ± 10%, *p* < 0.01). The Hb response shows a similar trend in the curve (Fig. 2e), however only the reoxygenated level showed a significant decrease in T1 in comparison to both room air (− 4 ± 3%, *p* = 0.02) and the deoxygenated state (*p* = 0.03). Moreover, chemical reduction did not impact T1 measurements for the Hb samples (− 1 ± 3%, *p* > 0.99 vs. room air).

**Figure 3:**
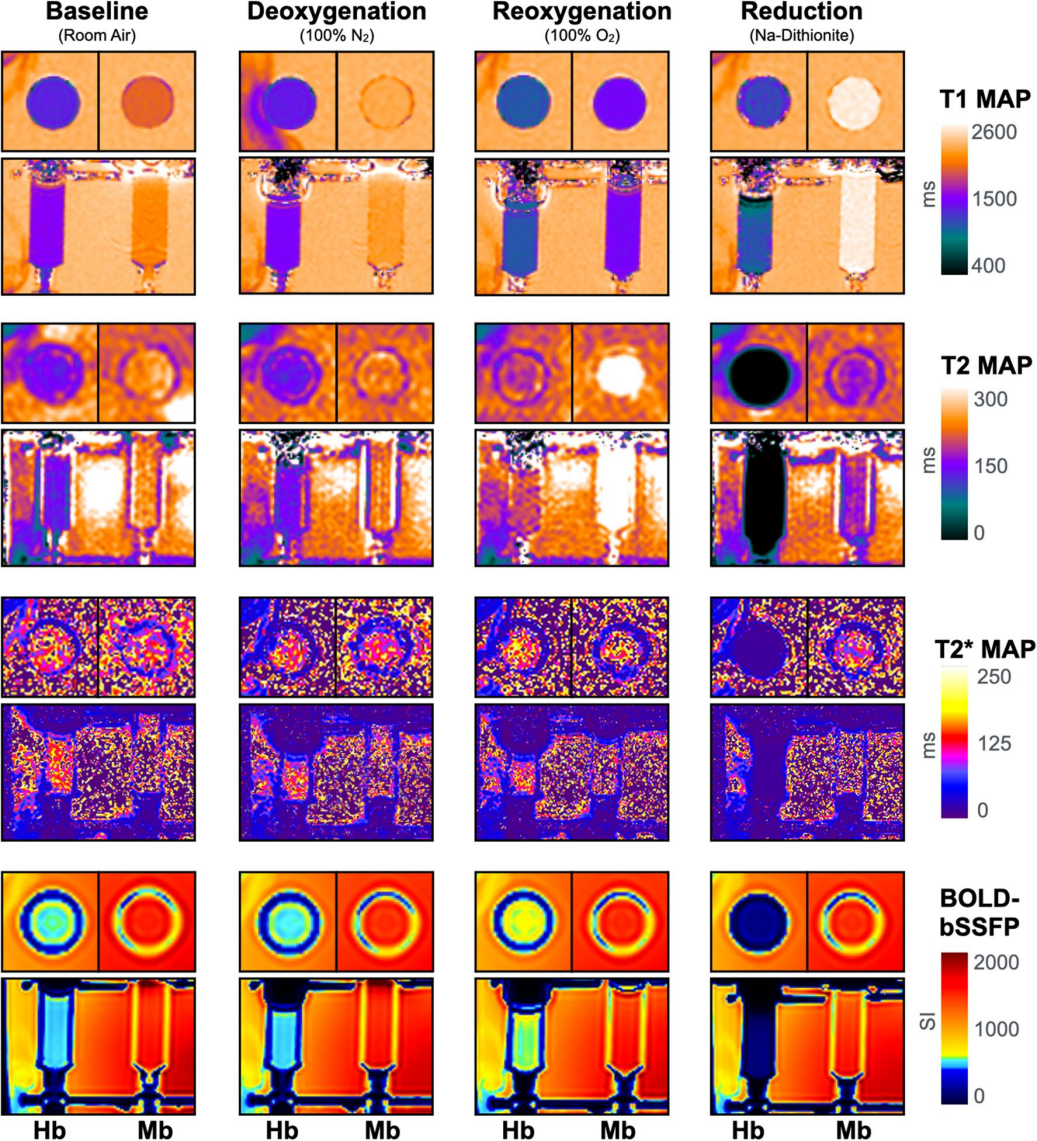
MRI imaging of myoglobin and hemoglobin: Horizontal and vertical cross-section views are shown of representative T1 maps, T2 maps, T2* maps and blood oxygen level-dependent balanced steady state free precession (BOLD-bSSFP) of red blood cells and dissolved myoglobin in modified test tubes at each major oxygenation state. On the left side a hemoglobin sample is shown, while on the right side a myoglobin sample is shown within the same imaging plane and for all acquired imaging sequences, respectively. Image prepared using Circle cvi^42^ version 5.13 (www.circlecvi.com).

Changing the oxygenation state of the specimens resulted in the opposite effect on transverse (T2) relaxation times, when compared to T1 mapping (Fig. 2b,f). T2 mapping of Mb samples showed T2 shortening after deoxygenation with N_2_ of − 11 ± 7% (*p* < 0.01), a subsequent prolongation after reoxygenation with O_2_ of + 17 ± 10% (*p* < 0.01 vs. room air and deoxygenation), and finally the strongest decrease in T2 after reduction with sodium-dithionite (− 28 ± 5%, *p* < 0.01 vs. all levels). With Hb samples, deoxygenation did not alter T2 relaxation time from room air (− 3 ± 5%, *p* > 0.99), however reoxygenation significantly increased T2 above the room air (11 ± 5%, *p* = 0.01) and deoxygenated samples (*p* < 0.01). Chemical reduction had a profound effect, decreasing signal to − 82 ± 1% (*p* < 0.01). Images for the key oxygenation levels can be seen in Fig. 3.

### T2* and BOLD-bSSFP

Deoxygenation by N_2_ significantly shortened T2* in Mb samples by − 12 ± 8% (*p* = 0.02, Fig. 2c) from baseline, which then normalized again with reoxygenation (0.3 ± 7%, *p* > 0.99 vs. baseline, *p* = 0.01 vs. deoxygenation). No changes were observed with the chemical reduction (Fig. 2c). With Hb deoxygenation (− 6 ± 4%) shortened T2* as well below both room air (*p* = 0.04) and reoxygenation (2 ± 3%, *p* = 0.86 vs. room air, *p* < 0.01 vs. deoxygenation). Similar to T2, chemical reduction strongly reduced T2* in Hb (− 89 ± 1%, *p* < 0.01, Fig. 2g).

Finally using a BOLD-bSSFP, a sequence based on T1 and T2, no changes in signal were observed from room air, but chemical reduction (− 3 ± 6%) significantly decreased signal in Mb in comparison to the reoxygenated state (*p* < 0.01, Fig. 2d). In the Hb samples, de-oxygenated blood (− 0.7 ± 1%) did not differ in signal to baseline, however reoxygenation (8 ± 6%, *p* = 0.05 vs. room air) significantly increased the signal above the deoxygenated state (*p* = 0.02). BOLD-bSSFP signal intensity decreased 57 ± 2% with chemical reduction by sodium dithionite (*p* < 0.01, Fig. 2h).

## Discussion

BOLD-MRI and Mb-magnetic resonance spectroscopy (Mb-MRS) studies have been performed in humans, interrogating the skeletal muscle^13,17–19^. While Mb-MRS can specifically interrogate dMb concentration, BOLD-MRI studies however, were not designed to specifically target Mb. The BOLD-effect of Mb cannot be reliably separated from the BOLD-effect of Hb, as long as Hb is present. In this experimental Hb-free in-vitro sample, we were able to show that changes in oxygen content in the isolated Mb-specimens led to changes in mapping of transverse relaxation times (T2 and T2*), while T1 mapping yielded opposing changes. Additionally, we could confirm the known BOLD effects of Hb using modern imaging sequences with this in-vitro system. The observed deflections of signal intensities and relaxation times showed the same trajectories in Hb and Mb samples for these mapping sequences. It seems thus likely that isolated Mb exhibits a BOLD-effect analogous to Hb.

Light spectroscopy clearly showed distinct optical density spectra of the different Mb species, which are also described in other previous studies^11^. The following desaturation and resaturation confirmed the functional patency of the molecule to bind and release molecular oxygen and it indicated that the preparation process, i.e. dissolving, chemical reduction of metMb to dMb and desalting, was successful, leaving auto-oxygenated MbO_2_ at ambient room air. It is important to stress why an in-vitro approach was utilized to investigate the relaxation time and SI changes to different oxygenation states of Mb. Mb has a very low p_50_, which allows it to auto-oxygenate, even at room air, but this also means it is difficult to desaturate in-vivo unless very extreme physiologic conditions occur^11^. In skeletal muscle, Mb is prone to desaturate more frequently under exertion training or limited blood supply under anaerobe conditions^12,13^. In the myocardium, pO_2_ levels sufficiently low for Mb desaturation would likely result in severe tissue ischemia. In in-vivo imaging exams, the presence of Hb would greatly confound the results when trying to interrogate Mb oxygenation with a BOLD-like approach. Indeed, when both species, Hb and Mb, are present, we would see decreasing T2 and T2* as a result of deoxygenating Hb when lowering pO_2_ levels, while Mb would remain saturated. Klarhöfer et al. showed decreasing T1 and T2* in skeletal muscle during ischemia in human calf muscle at 1.5 T, however this does not discern Hb from Mb effects^19^.

Therefore, as a first proof of concept a Hb-free environment was required to establish if there is a BOLD-effect for Mb at all. The custom-made sample tubes were sealed to the ambient air with valves to prevent contamination with room air and thus auto-oxygenation of Mb and Hb after bubbling with N_2_. The increase in T1 mapping after bubbling N_2_ shows a depletion of freely dissolved O_2_. T1 was lower at both oxygenated states, i.e. at room air and after bubbling O_2_. It is known that molecular oxygen exerts paramagnetic effects on the solvent, which explains the decrease in T1 in the oxygenated state^20–22^. This, however, is in contrast to the T2 and T2* mapping results. In Mb in comparison to reoxygenated states, a decrease in oxygenation resulted in a shortening in T2 and T2*, which are susceptible to the paramagnetic effects of dMb. This effect is also known for Hb^2,3^ and can be seen in the samples containing Hb. In our model, the transition from the diamagnetic MbO_2_ to the paramagnetic dMb will alter the bulk magnetic susceptibility of the solvent water, which leads to these observed changes in T2 and T2*. The paramagnetism of dMb far exceeds that of molecular O_2_, which explains why we see these opposing effects in longitudinal (T1) in contrast to transverse relaxation (T2 and T2*) measurements, which are known to be BOLD-sensitive. The changes in T1, T2 and T2* can also be seen in Hb samples, albeit to a relatively lesser extent. There are multiple potential reasons for this attenuation: Firstly, it is known that Hb concentration impacts the measured oxygenation response in sequences susceptible to the BOLD-effect^23^. This is an inherent confounder of the measurement technique itself. Secondly, the total oxygen content bubbled through the sample may have only been sufficient to oxygenate a fraction of the Hb molecules. Hb molecules consist of a tetramer, while Mb is a monomeric compound. Thus, it is expected that more molecular oxygen is required. This could explain the attenuated relative increase in T2, T2* and SI in the bSSFP sequence compared to the Mb samples. This is partially supported by the diminished increase in T1 in the Hb samples, indicating that there is less chemically dissolved oxygen in the sample, as Hb is required to be fully saturated before oxygen will physically dissolve, based on the solubility index of oxygen. In addition, the weaker affinity of Hb to oxygen in comparison to Mb could also be a reason for the diminished response of Hb samples.

Van den Boomen et al. recently used a combined T2–/T2*-mapping approach over the course of a breath-hold to determine the BOLD responses between cardiovascular patients and healthy controls^24^. T2* mapping is more sensitive to magnetic field inhomogeneities and thus the BOLD-effect than T2 mapping techniques. The same applies to T2* versus T2 weighted imaging sequences. The latter employs refocusing pulses in order to correct for such field-inhomogeneities, which leads to decreased sensitivity to changes in tissue oxygenation. Importantly, T2 mapping is also capable of assessing the oxygenation status of Hb and has thus also a BOLD-effect. Varghese et al. used parametric T2 mapping to quantitatively assess blood oxygenation of the left and right ventricle^25^. Their findings are reflected in our results, where T2 is highly suggestive of a BOLD-like effect for dHb and dMb. Although T2* mapping might be more advantageous to measure changes in tissue oxygenation, it is also more challenging and our in-vitro results should be interpreted with caution. In our samples, we observed T2* was also compromised by the presence of residual gas mix at the top of the bubbling system that created artifacts, which were not observed with the other sequences acquired in the same imaging plane. In the heart imaging issues with T2* are mostly seen with susceptibility artifacts and lower T2* relaxations times in myocardium at the heart lung interface^26,27^. Consequently, only the septum is investigated for pathologic T2* relaxation times in clinical scans. T2* mapping, especially at higher field strength, is also much more sensitive to artifacts. This could be seen in the higher exclusion rate of T2* maps at 3 T in our experiments. This is why it is currently still recommended to be performed only at 1.5 T clinically^27^. It is expected that the relative changes in T2* should be more pronounced than those of T2 mapping with changing oxygenation states. This is founded by the sensitivity of local field inhomogeneities in T2* transverse relaxation times in addition to the sensitivity to spin–spin interaction in T2 mapping^3,27^. It would be of interest for future studies to investigate the relationship between Mb and T2* imaging at 1.5 T as well.

The BOLD-bSSFP sequence has also been implemented in recent studies to assess the oxygenation response of the myocardium^28–30^. The observations in our red blood cell samples support the BOLD effect of Hb on bSSFP as we observed that oxygenated Hb samples had a higher signal than in images where dHb was deoxygenated by either nitrogen gas or chemical reduction. However, we were not able to demonstrate significant changes in BOLD-SI of the myoglobin samples between the deoxygenated and oxygenated states, although a detectable drop in SI was observed when the sample was reduced with dithionite in comparison to oxygenated level. Balanced SSFP sequences show a BOLD-effect, but are characterized by a T2/T1 contrast^31^. In our study, we saw reciprocal changes in T2 versus T1 in the Mb samples, which likely attenuated the changes in overall SI between the oxygenation steps for the bSSFP sequence approach. On the other hand, Hb samples did not have a profound T1 effect, and thus there was less of a competing balance between the T1 and T2 effects and consequently significant effects were observed with Hb and T2*.

Mb is known to play a major role in skeletal muscle fibers. Mb with its higher oxygen affinity compared to Hb allows the muscle cell to quickly accept the O_2_ from the red blood cells^11,15^. Thus, its main purpose is to facilitate O_2_-diffusion^32^ and O_2_ storage^33^. The striated muscle highly relies on Mb oxygen diffusion and storage in high intensity training and in restricted blood supply^12,13^. However, the role of Mb in myocardial function is more controversial: Mb-deficient knock-out mice showed severe cardiovascular defects in-utero, which resulted in death^14^. Yet, Mb-deficient knock-out mice that survived showed no significant difference in sarcomere morphology and mitochondrial count. However, they showed some cellular and metabolic compensatory mechanisms, such as higher capillary density, higher myocardial blood-flow and a higher hematocrit^14,34,35^. Endeward et al. reported a study, in which their group used a Krogh cylinder model to demonstrate that with a heart rate of 200/min in humans, the duration of diastole is shortened so that both systole and diastole have the same duration (150 ms)^15^. In this case, it is highly likely that Mb-facilitated O_2_-transport secures cellular oxygen supply to the left ventricular wall during cessation of blood flow during systole for 22–34 ms^16^, followed by dependence on the O_2_-storage function 12–17 ms. If Mb was absent, cellular anoxia would likely occur after 116–99 ms. The authors conclude that while Mb plays no role during diastole, in this setting it supplies O_2_ to the left ventricular wall for up to one third (50 ms) of the 150 ms systole, whereas capillary Hb is responsible for up to 80 ms^15^. It is important to note that Hb will firstly desaturate before Mb can do so. It is clear that approaches exploiting the BOLD-effect can detect these changes in Hb desaturation, and this is observed as well in our in-vitro Hb samples with the T2, T2* and BOLD-bSSFP images. Our data suggest that the subsequent desaturation of Mb could be detectable as well. Importantly, the effects that Endeward et al. reported apply to the healthy human heart^15^. Patients with a restricted flow reserve or in other pathologies in which oxygen demand may outweigh the supply, i.e. coronary artery disease, heart failure, hypertrophic cardiomyopathies, it can be expected that this effect may in fact occur at much lower heart rates, when patients feel exertion. Guensch et al. showed in a swine model that BOLD-SI only drops when oxygen demand outweighs the supply^36^. In patients with cardiovascular disease, this balance may be much more delicate and shifted, with fewer reserves on the supply side. However, previous publications were not designed to show Mb-specific effects. Recent cardiovascular MR studies used BOLD-sensitive cines that have sufficient temporal resolution to detect oxygenation deficits in systolic and diastolic cardiac phases to test such a theoretical paradigm postulated by Endeward and colleagues^15^. It remains to be seen if BOLD-MRI measurements targeting Mb saturation as the ultimate tissue oxygenation marker will have a potential clinical role in the future. Such studies could distinguish a decrease in extracellular oxygenation reserve driven by Hb deoxygenation from genuine tissue ischemia driven by intracellular depletion of Mb oxygen stores. This may serve as a future severity marker for ischemic heart disease. This concept would however require further experimental validation in muscle samples and eventual in-vivo studies.

The proportional role of Mb and Hb may also depend on the amount of protein in the imaging plane. Our in-vitro results are depicted as a %-change from the room air level to account for differences in concentration of the Mb and Hb proteins in each sample. In an in-vivo environment, the concentration of these proteins in an imaging plane will vary as well. In a study by Guensch et al. the authors showed that lowering the Hb concentration in-vivo increases baseline SI and attenuated the oxygenation response exploited induced by vasoactive stimuli^23^. Myoglobin is present in skeletal muscle at approximately 400–500 µmol/kg of wet mass tissue and at about 200–300 µmol/kg in myocardial tissue^37^. With a molar mass of 17,600 g/mol, the equine Mb used in this study is equivalent to the human Mb protein^37^. While muscle density differs on the muscle and patient health, generally skeletal and myocardial muscle have a density around 1.055 g/ml, which yields an approximate concentration range of 6.67–8.34 mg/ml of myoglobin protein content in skeletal muscle and 3.22–5.00 mg/ml in the myocardium. In our samples, the measured Mb concentration ranged from 4.24 to 4.42 mg/ml and is in line with the expected concentration in the myocardium. While Mb is primarily fixed in the muscle, it does circulate in the blood bound to plasma globulins, but at a minimal level (< 0.03 mg/ml)^38^. Mb can be released in higher concentrations due to muscle injury. Stone et al. reported a mean serum Mb of 528 ± 76 ng/ml after acute myocardial infarction^39^, while Wasfie et al. showed Mb can increase further in trauma patients with a high injury severity score reporting a maximum measured value of 11,197 ng/ml^40^. It is unknown how this would impact MRI signal and relaxation as the concentration of serum Mb is still relatively low in comparison to the Mb content of the muscle and to hemoglobin. And due to its higher affinity to oxygen, it is unlikely to desaturate in the blood stream, not even in venous blood. Thus, released serum Mb is unlikely to confound studies exploiting the BOLD-effect of Hb.

We used a Hb concentration between 10.0 and 11.0 g/dl, which is slightly lower than the general human reference ranges for arterial blood (12.0–18.0 g/dl). However, in an in vivo model the local Hb content in the imaging plane can fluctuate based on arteriolar vasodilation and capillary recruitment throughout the tissue. Capillaries, in particular, are the vessels that contain the majority of blood volume and dHb and thus have a high impact on the BOLD effect^9^. In addition, as the Hb molecules were inside intact red blood cells, a certain degree of sedimentation may occur. However, this may have only played a minor role, as the red blood cells would have been mobilized through the bubbling process and image acquisition took place within a couple minutes, before the next bubbling occurred. Thus, significant sedimentation is unlikely and was not visually observed in the samples.

There are limitations associated with this work. This proof of concept required an experimental in-vitro model, where no confounding Hb was present, therefore we chose to use lyophilized equine Mb powder. This model possesses both, strengths and weaknesses: first, by lacking other cellular proteins that may exert paramagnetic effects as well, we can be sure that the observed effects result from Mb oxygenation changes alone. However, these conditions make it harder to be translated into a cellular in-vitro and in-vivo environment. Nor can these isolated measurements determine how much Mb will contribute to the BOLD effect in an in-vivo model. As observed in the red blood cell samples, chemical reduction by sodium dithionite resulted in a significantly reduced signal beyond that observed in Mb. Moreover, a significant difference between the in-vitro and in-vivo environments is that the in-vivo myocardial oxygenation balance is influenced by perfusion changes and oxygen consumption of the tissue, whereas our in-vitro assessments do not incorporate these factors. Increased tissue perfusion will result in an increased MRI signal in BOLD-sensitive approaches due to a higher blood volume increasing the local content of bulk water and due to an increased oxygen supply to the tissue, resulting in the oxygenation of Mb and Hb and the washing out of dHb. Rather, the in-vitro assessments simplify the BOLD response by eliminating potential confounders based on physiologic responses and demonstrate if the paramagnetic properties of these compounds do impact MRI signal based on the oxygenation changes alone. A direct comparison of our measurements with Mb-MRS would have strengthened our model. Unfortunately, MRS was not available. However, since there was no other molecule in this in-vitro model, and desaturation during light spectroscopy verified desaturation, it is very suggestive that bubbling 50 ml of 100% N_2_ through a 5 ml sample voided the sample of oxygen and induced Mb desaturation. It was not possible to determine the oxygen saturation of Mb and Hb or partial pressure of oxygen pO_2_ with a blood gas analyzer because obtaining the sample would have causes air contamination, resulting in auto-oxygenation of Mb and falsely high pO_2_. T2* mapping showed the same trends as T2 mapping with Mb oxygenation changes. We would have expected the changes in T2* to be more pronounced. The fact that T2 mapping also showed the BOLD-effect for Mb with much better image quality makes this approach a better candidate for assessing Mb oxygenation changes at 3 T than T2* mapping. In the future the same measurement should be performed with different Mb concentrations and oxygenation states to further corroborate our findings. In a study assessing different Hb concentrations in-vivo, it has been shown that hemodilution has profound effects on BOLD-CMR measurements^23^.

Our data demonstrate that T2 and T2*-shortening after deoxygenation of in-vitro Mb specimens, which is highly suggestive of a BOLD-like effect, similar to that of Hb in red blood cells. The changes in oxygen concentration in the samples are supported by inverse changes in T1 mapping. Further studies are warranted and required to corroborate this concept, to assess the overall contribution of Mb, and to determine if a potential clinical utility exists for Mb.

## Methods

### Myoglobin preparation

**Figure 4:**
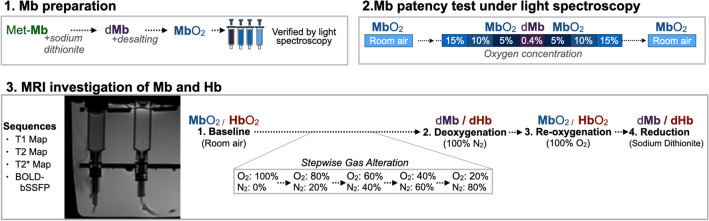
Experimental Procedure: (1) Equine metmyoglobin (metMb) was first reduced to deoxymyoglobin (dMb) and desalted to gain oxygenated myoglobin (MbO_2_) by auto-oxygenation, (2) after which samples were verified with light spectroscopy during decreasing then increasing oxygen concentrations. (3) Myoglobin solution and packed red blood cells were then placed into sealed compartments designed for gas bubbling. MRI was performed with four sequences in a vertical view, and one horizontal cross-section view under conditions designed to oxygenate and de-oxygenate myoglobin (Mb) and hemoglobin (Hb).

Commercially available lyophilized equine metmyoglobin (metMb) powder (Sigma-Aldrich, St. Louis, USA) was dissolved in PBS-buffer to yield a concentration of 20 mg/mL (Supplemental Figure 1). MetMb, myoglobin with Iron(III), does not bind oxygen^41^. Therefore, samples were reduced by addition of sodium-dithionite to dMb, now containing Iron(II). Sodium-dithionite will result in a reduction of pH, thus pH was adjusted to 7.35–7.45 to ensure normal function of Mb. As dMb cannot oxygenate in the presence of residual sodium-dithionite ions, the excess sodium-dithionite was removed from solution with a separation column (GE Healthcare HiPrep 26/10 Desalting column, GE Health Care, Chicago, USA) according to the vendor’s directions and as published before^11^. This allowed for auto-oxygenation of Mb at room air (MbO_2_) due to the very low p_50_. During the desalting process aliquots were sampled every 30 s, which were then interrogated with light-spectroscopy for the presence of dMb and thus residual sodium-dithionite (Fig. 4). Samples with pure MbO_2_ was used for further analyses and samples with dMb were discarded due to high probability of residual sodium dithionite.

### Analysis for functional myoglobin patency

In the next step the functional capability of Mb molecules was interrogated, verifying MbO_2_ could desaturate to dMb by depriving the samples from oxygen (O_2_). The MbO_2_ samples were pipetted into the wells of a microplate reader for light spectroscopy (Tecan Infinite 200 Pro, Tecan Group Ltd, Switzerland). Optical density (OD) was measured in 10 nm increments between 500 and 700 nm wavelengths. This was performed at room air (20% O_2_) and repeated at 15%, 10%, 5% and finally 0.4% environmental oxygen in the plate reader. The remainder of the partial pressure was driven by supplemental nitrogen (N_2_). Thereafter, oxygen concentration was restored in reverse steps.

### Hemoglobin preparation

Hb was assessed with the use of blood samples. Human packed red blood cells, which were not to be used in recipients anymore, were acquired from the central blood lab at the institute and diluted by a factor of two with saline to achieve a Hb concentration between 100 and 110 g/L.

### Imaging and analysis

The Mb and Hb samples were then transferred in custom-made 5 ml compartments that were sealed off by inlet and outlet valves for bubbling gases through the samples and allow for pressure equalization. An additional Luer-Lock hub was used to bubble nitrogen (N_2_) and oxygen (O_2_) through the samples and to add sodium-dithionite to conclude the experiments. All samples were scanned submerged in a water bath at room temperature and were imaged with a 20-channel head coil in a 3 T MAGNETOM Prisma Fit (Siemens Healthineers, Erlangen, Germany). Clinical sequences from the cardiac package were used and modified to obtain a higher spatial resolution. Images were triggered with a simulated heart rhythm with an RR interval of 1200 ms. Imaging parameters can be found in Table 1. At room air, T1, T2 and T2* maps along with BOLD-bSSFP images were scanned in an axial cross-section and vertical long-axis view of the specimen compartment. The same images were obtained at each level after the bubbling process had been completed. First a step-wise deoxygenation was performed by bubbling a mix of O_2_ and N_2_ through the specimen over 2 min. This was performed in 20% increments starting with (1) 0% N_2_/100% O_2_; (2) then 20% N_2_/80% O_2_; (3) 40% N_2_/60% O_2_; (4) 60% N_2_/40% O_2_; (5) 80% N_2_/20% O_2_; and (6) 100% N_2_ to obtain full deoxygenation. Afterwards samples were reoxygenated by bubbling 50 ml 100% O_2_ and finally chemically reduced by adding sodium-dithionite. Twelve Mb and Hb samples were imaged at all levels, with an additional ten Mb samples acquired at the key stages: room air, deoxygenation (100% N_2_), re-oxygenation (100% O_2_) and chemical reduction.

**Table 1:** Imaging parameters.

Sequence	T1 Map	T2 Map	T2* Map	BOLD-bSSFP
Voxel (mm^3^)	0.5 × 0.5 x 5.0	0.6 × 0.6 x 5.0	0.5 × 0.5 x 5.0	0.7 × 0.7 x 0.5
FOV (mm)	250 × 250	223 × 178	215 × 215	267 × 267
TE/TR (ms)	1.58/3.6	1.58/3.7	*/19.0	1.93/3.9
Flipangle (°)	35	12	15	35
Bandwidth (Hz/Px)	977	1184	503	1302
Averages	1	1	1	1
Specific Parameters	3(3)5 MOLLI, MOdified Look-Locker Sequence	Gradient echo Adiabatic T2-prep (0, 25, 55 ms)	Gradient echo *TE: 3.0, 5.5, 7.9, 10.4, 12.8, 15.3, 19.0	True-FISP

FOV: Field of View, TE: Echo time, TR: Temporal Resolution.

Images were analyzed with clinically validated software, cvi^42^ (Circle Cardiovascular Imaging Inc, Calgary, Alberta, Canada). For all images, regions of interest (ROI) were traced in the axial cross-section of a sample carefully avoiding artifact at the border of the compartments (Supplemental Figure 2). ROI were then copied and forwarded to remaining levels to ensure the same positioning and adjusted in cases of where the sample position had shifted. In the case of poor image quality, contours were also drawn in the vertical long-axis view and the data was then averaged.

### Statistical analysis

To account for different concentrations and thus baseline signal, data are shown as a %-change in signal from the first level acquired at room air (mean ± 95% confidence intervals). Measurements were statistically compared between the key oxygenation states (room air; deoxygenation with 100% N_2_; re-oxygenation with 100% O_2_; and chemical reduction) for both Mb and Hb with a mixed effects linear model. Bonferroni’s multiple comparisons tests were performed after, if applicable. Statistical results were regarded significant with a two-sided *p* < 0.05. For light-spectroscopy, only qualitative assessments were performed. GraphPad Prism version 8.0 (GraphPad Software, La Jolla California USA) was used for statistical analysis.

The datasets generated during the current study are available from the corresponding author on reasonable request.

## Supplementary Information

Supplementary Information.
