# Supplementary material for: The blood oxygen level dependent (BOLD) effect of in-vitro myoglobin and hemoglobin
Source: Sci Rep. 2021 Jun 1;11:11464. doi: 10.1038/s41598-021-90908-x (PMC8169704; doi:10.1038/s41598-021-90908-x)
Supplement: Supplementary file 1 — Supplementary Information. [file 41598_2021_90908_MOESM1_ESM.pdf]

SUPPLEMENTARY FILE

The Blood Oxygen Level Dependent (BOLD) Effect of in-Vitro Myoglobin and Hemoglobin

Dominik P. Guensch[1,2], MD; Matthias C. Michel[1], MD; Stefan P. Huettenmoser[2]; Bernd Jung[2], PhD; Patrik Gulac[3,4,5], PharmaD, Adrian Segiser[3,4], MSc; Sarah L. Longnus[3,4], PhD; Kady Fischer[1] PhD.

1) Department of Anaesthesiology and Pain Medicine, Inselspital, Bern University Hospital, University of Bern, Bern, Switzerland.
2) Department of Diagnostic, Interventional and Paediatric Radiology, Inselspital, Bern University Hospital, University of Bern, Bern, Switzerland.
3) Department of Cardiovascular Surgery, Inselspital, University Hospital Bern, Bern, Switzerland
4) Department for BioMedical Research, University of Bern, Bern, Switzerland.
5) Department of Pharmacology and Toxicology, Faculty of Pharmacy, Comenius University, Bratislava, Slovakia.

Running Title: BOLD-Effect of Myoglobin

Supplemental Figure 1: Myoglobin Preparation

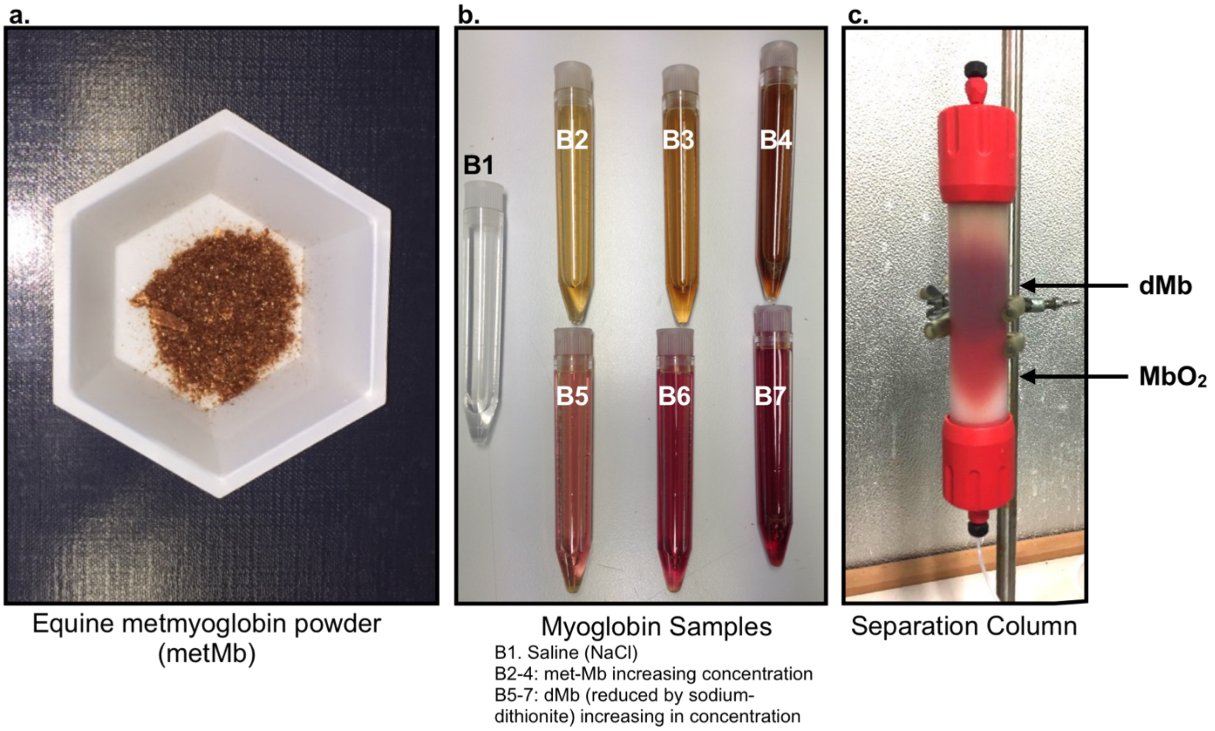

A. Commercially available lyophilized equine metmyoglobin (metMb) powder (Merck KGaA, Darmstadt, Germany).

B. MetMb was dissolved in PBS-buffer in increasing concentrations of metMb (top row) and converted into deoxygenated Mb (dMb, bottom row) by addition of sodium-dithionite (reduction from iron Fe^{III+} to Fe^{II+} in order to allow for binding), from which a maximum concentration of 20mg/ml met-Mb was used.

C. In the presence of sodium-dithionite, oxygenation is not possible. Thus, the excess sodium-dithionite was removed from solution with a separation column (GE Healthcare HiPrep 26/10 Desalting column, GE Health Care, Chicago, USA), which allowed for auto-oxygenation of Mb in room air (MbO_2). The transition from dMb to MbO_2 could be seen during the passage of the solution through the desalting column.

Supplemental Figure 2: MRI Analysis

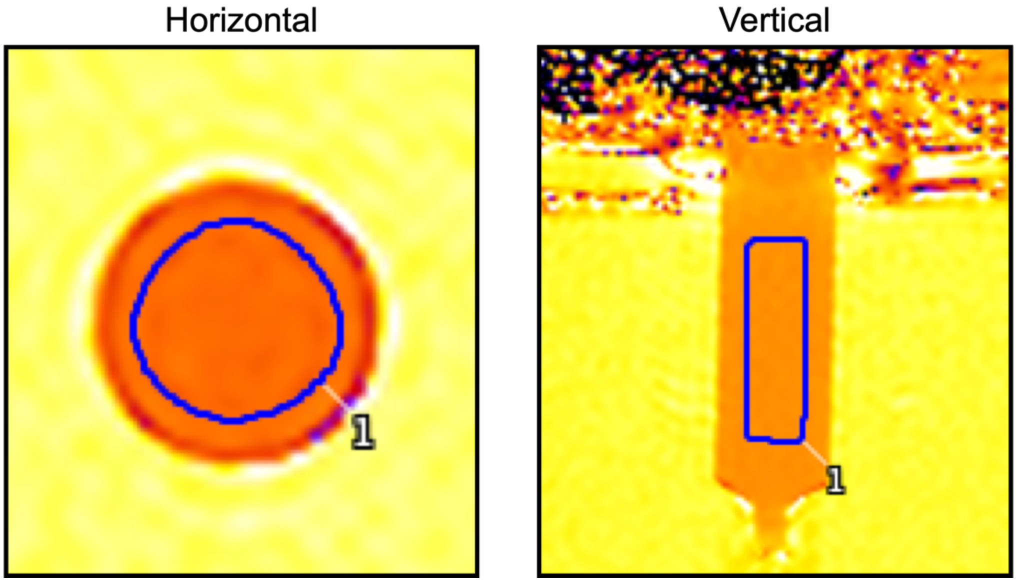

Regions of Interest (ROI) are depicted by the blue contour in a horizontal and vertical cross-section view of a T1 map. Image prepared using Circle cvi42 version 5.13 (www.circlecvi.com).
